# The TeleTriageTeam, Offering Continuity of Personalized Care Through Telemedicine: Development and Evaluation

**DOI:** 10.2196/46145

**Published:** 2023-07-28

**Authors:** Janneau Claessens, Sigrid Mueller-Schotte, Jeannette van Weerden, Helianthe Kort, Saskia Imhof, Robert Wisse

**Affiliations:** 1 Department of Ophthalmology University Medical Centre Utrecht Utrecht Netherlands; 2 Department of Optometry and Orthoptics HU University of Applied Sciences Utrecht Utrecht Netherlands; 3 Research group Technologies for Healthcare Innovations Research Centre Sustainable and Healthy Living HU University of Applied Sciences Utrecht Netherlands; 4 Xpert Clinics Oogzorg Zeist Netherlands

**Keywords:** triaging, telehealth, telemedicine, remote care, ophthalmology, eye care, mobile phone, COVID-19

## Abstract

**Background:**

The COVID-19 pandemic taught us how to rethink care delivery. It catalyzed creative solutions to amplify the potential of personnel and facilities. This paper presents and evaluates a promptly introduced triaging solution that evolved into a tool to tackle the ever-growing waiting lists at an academic ophthalmology department, the TeleTriageTeam (TTT). A team of undergraduate optometry students, tutor optometrists, and ophthalmologists collaborate to maintain continuity of eye care. In this ongoing project, we combine innovative interprofessional task allocation, teaching, and remote care delivery.

**Objective:**

In this paper, we described a novel approach, the TTT; reported its clinical effectiveness and impact on waiting lists; and discussed its transformation to a sustainable method for delivering remote eye care.

**Methods:**

Real-world clinical data of all patients assessed by the TTT between April 16, 2020, and December 31, 2021, are covered in this paper. Business data on waiting lists and patient portal access were collected from the capacity management team and IT department of our hospital. Interim analyses were performed at different time points during the project, and this study presents a synthesis of these analyses.

**Results:**

A total of 3658 cases were assessed by the TTT. For approximately half (1789/3658, 48.91%) of the assessed cases, an alternative to a conventional face-to-face consultation was found. The waiting lists that had built up during the first months of the pandemic diminished and have been stable since the end of 2020, even during periods of imposed lockdown restrictions and reduced capacity. Patient portal access decreased with age, and patients who were invited to perform a remote, web-based eye test at home were on average younger than patients who were not invited.

**Conclusions:**

Our promptly introduced approach to remotely review cases and prioritize urgency has been successful in maintaining continuity of care and education throughout the pandemic and has evolved into a telemedicine service that is of great interest for future purposes, especially in the routine follow-up of patients with chronic diseases. TTT appears to be a potentially preferred practice in other clinics and medical specialties. The paradox is that judicious clinical decision-making based on remotely collected data is possible, only if we as caregivers are willing to change our routines and cognitions regarding face-to-face care delivery.

## Introduction

### Background

The importance of high-quality remote care was emphasized when most elective hospital care was on hold during the COVID-19 pandemic. The number of regular face-to-face patient consultations were reduced to comply with government-imposed mobility restrictions. Initially, face-to-face in-hospital consultations were considered only when medically urgent. The capacity of our academic outpatient clinic reduced by 90% (from 300 to 30 visitors per day). Before the pandemic, the capacity of ophthalmic care in the Netherlands was already barely sufficient, with accessibility under pressure and ever-growing waiting lists [1]. Future projections offer little perspective, with an estimated increase in national health expenditures from 12.7% of gross national income in 2015 to 19.6% by 2060, owing to our aging society [2]. To address these immediate and future challenges, we conceptualized and executed a novel telemedicine approach, the TeleTriageTeam (TTT).

The TTT is an ongoing collaboration between the HU University of Applied Sciences Utrecht (HU-UAS) and the University Medical Center Utrecht (UMCU). In this approach, a team of undergraduate (ie, bachelor’s degree) optometry students, tutor optometrists, and ophthalmologists worked together to remotely provide eye care [3]. Although originally conceptualized for telephonic triaging and rescheduling appointments during the acute pandemic-related capacity crisis, the approach has evolved into a telemedicine service that included advising patients, refining treatment, or referring patients to other physicians. It appeared highly valuable beyond the acute crisis and is therefore still ongoing. In addition to allowing the continuation of care, the approach created a unique opportunity to continue the training of optometry students during the pandemic while respecting social distancing and quarantining.

### Objective

In this paper, we described a novel method of delivering remote care safely and effectively using an innovative approach to interprofessional task allocation and the application of technology for remote vision testing. We aimed to report on the clinical effectiveness of the TTT approach and its impact on waiting lists and discuss its transformation to a sustainable method for delivering remote eye care.

## Methods

### Synopsis

The TTT approach included evaluations of current (ocular) health status using semistructured anamneses by telephone conducted by optometry students. If visual acuity was of interest for clinical decision-making, patients were requested to perform a remote, web-based eye test in their home environment. Patients were called back after their cases had been discussed by the supervising ophthalmologists, who were responsible for the clinical decision-making.

### Process Overview

#### Eye Care Delivery Before the Pandemic

The UMCU is a tertiary clinic and training institution. Most of the patients in the ophthalmology department have complex and multifactorial eye disorders. New cases typically present after referral by ophthalmologists from regional clinics. After diagnosis and treatment, most of the patients will be referred back to the referring ophthalmologist or the general practitioner (GP) once the condition is stable. Exempts from this policy are complex cases in need of indefinite academic care. Teleconsultations that replaced in-office visits were fairly uncommon, and video consultations were not performed.

#### Eye Care Delivery During the Pandemic

When the COVID-19 pandemic began in March 2020, about 90% of our outpatient capacity had to be reduced, greatly impacting scheduled appointments and waiting lists. Teleconsultations (ie, telephonic or video-assisted consultations) were preferred to face-to-face in-hospital consultations to limit the number of hospital visitors. Patients were referred to the hospital only when medically urgent (eg, neovascular age-related macular degeneration, poorly regulated glaucoma, and retinal detachments). To help prioritize scheduled appointments and restructure the waiting lists in our ophthalmologic department, the TTT was conceptualized. This approach was continued throughout the pandemic, during the various stages of lockdowns and subsequent changes in social restrictions. Shortly after its introduction, TeleTriage became a part of the standard curriculum for the optometry training at the HU-UAS. After a 2-day training program that focused on navigating through the electronic health record (EHR), best practices in data handling, and patient communication, students were enrolled in the TTT program for 4 weeks.

#### TTT Workflow

**Figure 1:**
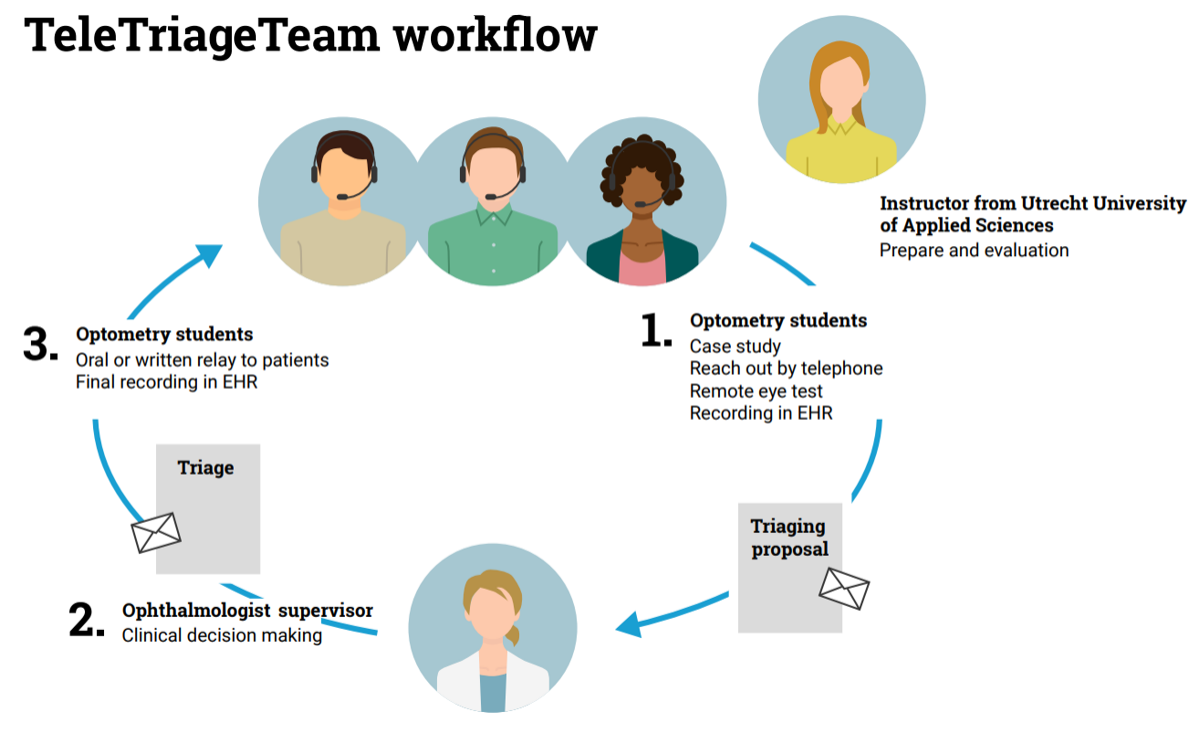
Workflow of the TeleTriageTeam. Optometry students reach out to patients by telephone and make a triaging proposal. A supervising ophthalmologist will make the final clinical decision. The patient will be informed by the students and the decision will be recorded in the electronic health record (EHR). A tutor optometrist will be on site for overseeing the process, assigning patients to the students, and prediscussing proposal options based on current guidelines.

Students of the TTT were assigned patients who were on the waiting list or scheduled for ophthalmology resident clinics. First, the students thoroughly studied and summarized the available information on the EHR. Subsequently, they reached out to the patients by telephone for semistructured anamnesis. A triaging checklist was used to assess the current eye health status and identify any changes in general health or medication use. The primary learning task for the optometry students was to make a triaging proposal based on the gathered information, adhering strictly to the existing clinical protocols. The students were supervised by a qualified tutor optometrist and an ophthalmologist. Under Dutch law, the ophthalmologist is responsible and accountable for the final clinical decision. Triaging decisions for the clinical appointment included the following options: maintain, expedite, postpone, cancel, change into a telephone or video consultation with their physician, or refer regionally. Case summaries were recorded in the EHR, and clinical decisions were relayed back to the patients and, if applicable, to the patients’ GPs or the referring ophthalmologists. Figure 1 depicts the workflow of the TTT.

#### The Remote Eye Test

In some cases, patients were requested to perform a remote eye test. This web-based Conformité Européenne (CE)–certified application enables individuals to self-assess their visual acuity in their home environment using their own electronic devices. This test was developed by Easee BV in collaboration with the UMCU and extensively studied in various patient populations [4-7]. To perform it at home, patients need an internet connection, a smartphone, and a computer or tablet. After entering the test via a website on their computer or tablet, users will be instructed to connect their smartphone by scanning a QR code or entering a code sent by an SMS text message. The patients are instructed to stand or sit 3 m from their screen and cover one eye with their hand while the computer or tablet screen displays a sequence of optotypes that the patient should correctly identify. A calibration step ensures that the displayed symbols are correctly sized, regardless of the screen dimensions of the user’s own devices. The smartphone is used as a remote control to submit the answers. At the end of the test, a visual acuity score will be presented (in Snellen decimal system, the common notation to express this outcome in our clinic).

The remote eye test was available via our clinic’s patient portal website. All patients of our clinic have direct access to their medical records via this secured web-based portal. Access is granted through a government-backed identification system (“DigiD”) [8], which ensures data safety and privacy of this digital environment. Patients were directed to the portal to open the eye test via a web link and instructed to write down or save their eye test results after completion. Within the portal, a dedicated questionnaire allowed patients to report their outcomes, after which it became available to the health professionals in the EHR. This manual step was required because the data were not automatically transferred between the remote eye test and the EHR.

### Study Population

This study database included all patients who were assessed by the students as part of the TTT project between April 16, 2020, and December 31, 2021. In principle, all patients on waiting lists or with scheduled appointments at the general resident outpatient clinic of the UMCU were screened for eligibility for teletriaging. Patients scheduled for a subspecialty appointment (eg, patients with uveitis and patients referred to pediatric ophthalmology or vitreoretinal consultants) were excluded from the project because of the anticipated complexity of the cases. The consultant ophthalmologists were responsible for downscaling their waiting lists, and these cases are not covered in this paper. Ophthalmologists specializing in corneal pathology were involved in supervising the TTT (depicted in Figure 1 as “ophthalmologist supervisor”); hence, a minority of cases were considered subspecialty cases from the corneal clinic. No further exclusion criteria were applied.

### Data Collection

We used real-world clinical data and demographics of the TTT project, gathered by the optometry students, registered in Microsoft Excel (version 16.0.4266.1001 for Windows; Microsoft Corp) and the EHR, HiX (version 6.1; Chipsoft). The characteristics included in the database were as follows: age, sex, diagnosis, date of triaging contact, reachability by phone (yes or no), possibility of a video consultation (yes or no), remote eye test offered (yes or no), remote eye test performed (yes or no), triage proposal by the student, and final decision by the ophthalmologist. Free-text variables were recoded into categories before the analysis. Business data on waiting lists and patient portal access were collected from the capacity management team and IT department of our hospital.

### Data Analysis

The outcomes of this study included the clinical characteristics of the assessed patients, triaging decisions, uptake of the remote eye test, and the effects of triaging on the waiting lists and case mix of our outpatient department. The TTT project had an iterative development to optimize the service. Therefore, interim analyses were performed at different time points during the project, as part of the scheduled project evaluations. This paper presents the synthesis of these analyses.

Statistical analyses were performed using the SPSS Statistics (version 25; IBM Corp). Demographic data, clinical characteristics, and triaging outcomes were available for all included patients (April 16, 2020, to December 31, 2021). These data are descriptively presented as frequencies and percentages and as means and SDs.

Data on patient portal access and uptake of the remote test were available for all patients included up to May 7, 2021. The data are descriptively presented as frequencies and percentages. The differences in age between active and nonactive patient portal users, invited and uninvited for the remote eye test, and successful and unsuccessful performance of the remote eye test were compared using the independent samples 2-tailed *t* test. Age differences between the groups were considered statistically significant at a *P*<.05.

### Ethical Considerations

An anonymized, coded version of the TTT database was used to analyze the clinical data, precluding the research team from tracking patients on an individual level. Analyses were performed in accordance with Dutch privacy laws and the Declaration of Helsinki in the context of quality control and health care evaluation. According to national regulations (Centrale Commissie Mensgebonden Onderzoek), ethics approval and informed consent are not required when the quality of a novel health care delivery system is investigated for local applications [9].

## Results

### Population Characteristics

Our database included 3658 registrations of cases that were assessed in this project. The clinical characteristics and demographics of the assessed patients are summarized in Table 1, and this distribution reflects the general outpatient clinic population of our academic hospital. Sex distribution among the patients was equal (female patients: 1902/3658, 52%). The mean patient age was 59 (SD 19) years. The most frequent diagnosis categories were “corneal and conjunctival diseases” (632/2527, 25.01%), “glaucoma” (432/2527, 17.1%), and “screening for ophthalmic disease” (322/2527, 12.74%). The latter included routine screening of patients who had systemic diseases and used chronic medication with an increased risk of ocular disease (eg, protocolled hydroxychloroquine screening).

**Table 1:** Demographics and clinical characteristics of patients assessed by the TeleTriageTeam.

			Values
**All cases (N=3658)^a^**			
	**Sex, n (%)**		
		Male	1756 (48)
		Female	1902 (52)
	**Age (years)**		
		Value, mean (SD)	59 (19)
		Value, range	11-97
**First-year cohort (n=2527)^b^**			
	**Diagnosis category^c^, n (%)**		
		Corneal and conjunctival diseases	632 (25.01)
		Glaucoma	432 (17.1)
		Screening for ophthalmic disease	322 (12.74)
		Screening for diabetic retinopathy	269 (10.65)
		Cataract and other lens abnormalities	266 (10.53)
		Retinal and macular diseases	225 (8.9)
		Eye lid and orbit pathologies	120 (4.75)
		Neuro-ophthalmological diseases	110 (4.45)
		Other (eg, refractive errors or unspecified vision loss)	97 (3.84)
		Uveitis	88 (3.48)
		Pathologies of the bulbus, sclera or vitreous	75 (2.97)

^a^All consecutive cases assessed between April 16, 2020, and December 31, 2021.

^b^All consecutive cases assessed between April 16, 2020, and April 7, 2021.

^c^Diagnosis categories are based on “Diagnosis Treatment Combinations”. The Diagnosis Treatment Combinations coding is the Dutch registration method for charging health care to the insurer or the patient.

### Triaging Outcomes

The triage outcomes are presented in Table 2. For approximately half (n=1789, 48.91%) of the 3658 assessed cases, an alternative to the conventional face-to-face consultation was found. The appointment was cancelled in 212 (5.8%) of the cases, or postponed 733 (20.04%) times, with a mean delay of 6 (SD 4) months. Of the total 3658 patients, the face-to-face consultations of 132 (3.61%) patients were changed to teleconsultations with the ophthalmologist. A substantial proportion of patients (492/3658, 13.45%) was dismissed from academic care, as there was no solid ground for specialized follow-up. Other decisions included consulting with other specialists (194/3658, 5.3%).

**Table 2:** Triaging outcomes based on the final clinical decision made by ophthalmologists.

Triaging outcomes	All cases (N=3658)^a^, n (%)
Consultation unchanged	1869 (51.09)
Consultation postponed^b^	733 (20.04)
Consultation expedited	26 (0.71)
Consultation cancelled	212 (5.8)
Consultation changed to teleconsultation	132 (3.61)
Referral to regional hospital or general practitioner	492 (13.45)
Other	194 (5.3)

^a^All consecutive cases assessed between April 16, 2020, and December 31, 2021.

^b^Mean delay 6 (SD 4) months.

The other half (1869/3658, 51.09%) of the patients still required the scheduled face-to-face examination at the clinic and were marked as “maintain the consultation.” The consultations of a few patients (26/3658, 0.71%) were expedited after noting warning signs in the telephonic anamnesis.

### Access to the Patient Portal and Uptake of the Remote Eye Test

Interim analyses of patient portal access and remote eye test performance were conducted 1 year after the start of the project in May 2021. Most of the assessed patients (1667/2634, 63.3%) up to this date were “active” users of the patient portal, meaning they had logged on to this web service at least once. These active users were, on average, slightly younger than those who did not access (mean age 55, SD 18 years vs mean age 65, SD 18 years, respectively; *P*<.001). Patient portal use decreased with age; 75.7% (390/515) of the patients who were aged <40 years were active users, whereas only 32.1% (97/302) of the patients who were aged ≥80 years used the service, as shown in Table 3.

**Table 3:** Access to the patient portal and remote eye test, stratified per age category^a^.

	Age (years)						
	All categories (n=2634)	<40 (n=515, 19.6%)	40-49 (n=234, 8.9%)	50-59 (n=409, 15.5%)	60-69 (n=583, 22.1%)	70-79 (n=591, 22.4%)	≥80 (n=302, 11.5%)
Active users of the patient portal, n (% of category total)	1667 (63.3)	390 (75.7)	170 (72.6)	301 (73.6)	395 (67.8)	314 (53.1)	97 (32.1)
Invited to perform the remote eye examination, n (% of active users)	184 (11)	43 (11)	17 (10)	38 (12.6)	52 (13.2)	29 (9.2)	5 (5.2)
Successful completion of the remote eye test, n (% of invited patients)	82 (44.6)	19 (44.2)	7 (41.2)	19 (50)	23 (44.2)	14 (48.3)	0 (0)

^a^Cross-sectional analysis based on data from University Medical Center Utrecht IT department in May 2021. The patients who were not actively using the patient portal were, on average, older than the patients who had been using the patient portal (mean age 65, SD 18 years vs mean age 55, SD 18 years; *P*<.001).

Table 3 indicates that 11% (184/1667) of the active patient portal users were invited to perform the eye test. The eye test was offered if three conditions were met: (1) patients had access to the patient’s portal, (2) patients had access to a smartphone and computer or tablet, and (3) visual acuity was of interest in clinical decision-making. The reasons for not inviting patients to perform the test were not registered. Invited patients were only slightly younger than those who were not invited (mean age 53, SD 18 years vs mean age 59, SD 19 years; *P*<.001). Among the invited patients, there was no significant difference in age between the patients who successfully performed the eye test after the invitation and those who did not (*P*=.91).

### Waiting List Reduction at the Outpatient Department

**Figure 2:**
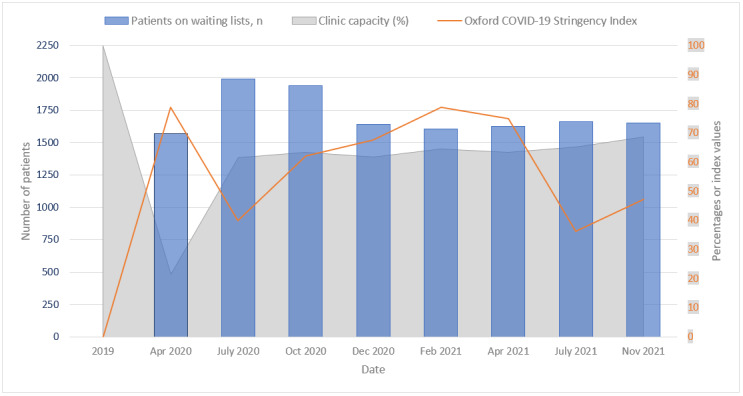
Number of patients on the waiting lists at the outpatient department of the ophthalmology residents and pandemic-related restrictions, as expressed by the clinical capacity (%) and Oxford COVID-19 Stringency Index. The total number of patients on the waiting lists is represented by the blue bars (left y-axis). The gray surface represents the operational clinic capacity (right y-axis), with 2019 data as the index (100). The orange line represents the Oxford COVID-19 Stringency Index (right y-axis), a composite measure based on 9 response indicators including school closures, workplace closures, and travel bans, rescaled to a value from 0 to 100 (100=the strictest).

To evaluate the impact of TeleTriage on our clinic’s health care delivery, we performed several cross-sectional investigations. Figure 2 illustrates the number of patients on the waiting lists in the outpatient department of the ophthalmology residents at our clinic and the pandemic-related restrictions, as expressed by the clinic capacity and lockdown stringency (as reported using the Oxford COVID-19 Stringency Index) [10]. The first blue bar effectively represents the prepandemic status of the waiting list, as the government’s lockdown measures were only effective from March 15, 2020, and waiting list effects needed some time to accumulate and materialize. During the following months, the waiting lists grew, peaking in summer 2020 (n=1991, July 2020). TeleTriage assisted tremendously in the continuation of the most urgent care because the clinic capacity was markedly reduced during this first lockdown (−80%). The lockdown stringency varied over time. Lockdown restrictions were lessened in summer 2020, coupled with a slight normalization of clinic capacity (approximately −30%).

The team productivity of the TTT peaked in July 2020, when approximately 500 cases were assessed over the month. Productivity mostly relied on the number of optometry students assigned to the project and the number of tutor optometrists. From September 2020 onward, TeleTriage became part of the standard curriculum of the optometry training at the HU-UAS, leading to a stabilized inflow of student optometrists. Naturally, other factors influenced the waiting list, such as the fluctuating number of new referrals or surgical capacity. Both dropped during the first months of the initial COVID-19 lockdown but normalized during 2020, albeit at a slightly lower level than in 2019 [11]. Since the end of 2020, we have managed to balance the influx of new patients and referrals to our general outpatient clinic with our reduced capacity and the added outflow of patients owing to TeleTriage, even with periods of imposed restrictions (as reflected by the increasing Stringency Index).

### Developing TeleTriage Into a Tool for Value-Based Health Care Delivery

During the initial global COVID-19 lockdown, TeleTriage served to retain continuity of care for the most urgent eye care. No patients with an urgent need for eye care were denied service within the TTT, including retinal detachments, progressive glaucoma, and wet age-related macular degeneration. Within months, our clinic’s capacity recuperated, and the TTT allowed us to process the backlog of patients awaiting an appointment (almost 2000 patients at its peak in summer 2020). Patients with lower urgency or complexity were often processed completely remotely and had their face-to-face consultations cancelled or postponed or were referred externally.

Interestingly, TeleTriage offered a potent means to judiciously select patients for nonspecialty follow-up with regional ophthalmologists or GPs. Referrals were customized to a high degree, with personal telephone feedback and a tailored written medical summary provided to both patients and caregivers. As a result, our patient population slowly but steadily grew more academic with less protocolled care of higher complexity.

A business analysis showed that the eye care delivered by our outpatient clinic in 2021 better adhered to the parameters of the academic care set in 2019. First, in 2021, registrations of Diagnosis Treatment Combinations fell significantly more often within our defined *academic care profile* (+15% increase compared with 2019). Second, the care delivered was significantly more often considered a *strategic theme* of the department (+14% increase compared with 2019). Our *academic care profile* is defined at the institutional level as tertiary referred care, pertaining to hospital-wide strategic themes, or last-resort care. *Strategic themes* are defined at the department level and indicate when certain Diagnosis Treatment Combinations are compliant with the vision of our management team and adhere to the spearpoints of the UMCU. Note that TTT only considered the general, glaucoma, and cornea outpatient clinics (25% of total patient volume) and not the other subspecialty clinics such as surgical, medical retina, uveitis or orbit, neuro-ophthalmology, and pediatrics. These analyses could only be made for our eye clinic as a whole, with an average of 8000 patients on the waiting list. Interestingly, the TTT still exerted a substantial effect on our overall case mix, whereas the addressable population was only 25% of our total clinic. Should one hypothetically apply this effect to all eye care patients, the case mix changes are assumed to be even more pronounced.

## Discussion

### Principal Findings

We present a novel method to triage eye care patients remotely, using interprofessional collaboration, teleconsultations, and remote vision testing. This project was conceptualized and catalyzed by the sudden COVID-19 pandemic. Subsequently, we developed it as a tool to deliver value-based health care beyond the primary pandemic setting. This innovation was successful in reducing approximately half of the planned care while providing continuity of care for the most urgent cases and deferring or cancelling consultations judiciously or referring the remaining patients after obtaining a specialist’s consideration. The TTT has helped mitigate the backlog of waiting lists that had been built during the initial months of the pandemic. Limited resources were required, and to the best of our knowledge, this telemedicine approach was the first of its kind to actively involve optometry students and remote eye testing in the workflow. Student participation is beneficial for teaching and training, but it can also enable a high turnover without additional staffing. To date, we continue to use this method in our department, as it offers a tool for value-based health care, delivering “the right care in the right place” (*“de juiste zorg op de juiste plek”* [12]), and is timely when relevant and needed. We propose that similar workflows could be conceptualized in other eye clinics with more GP referrals (eg, regular hospitals and specialized eye clinics), as less-complex pathology appeared easier to triage. However, this could be offset by a lack of available clinical data; in this project, we often had extensive patient charts at our disposal with numerous auxiliary investigations. Other medical specialties could similarly benefit from a working method as described here and contribute to the human capital challenges in both health care delivery and health care education, only if there is availability of technology for remote assessments and delegated personnel to interpret and collect these data [13-15]. Advanced eHealth technologies are not always required. Our project demonstrated that most triaging decisions were based on the clinical information collected by phone in addition to the data already available in the EHR.

When delivering remote care and triaging services, several ethical considerations and challenges should be considered. One major challenge is to deliver care that is noninferior to a face-to-face examination in terms of quality and safety. In this project, all optometry students worked under supervision, and none of their decisions were made independently. Although quality and safety aspects could not be examined in our descriptive analyses, we argue that in any form, clinical decision-making on available data by ophthalmologists is of the highest quality and pace when compared with other eye care professionals. Our asynchronous method, in which patients were called back after the case discussion with the supervising ophthalmologist, allowed ample time to thoroughly review patient health records and look into national and international guidelines and peer-reviewed literature. In addition to improving quality of care, this method also creates excellent teaching opportunities with real exposure to patient communication and clinical decision-making. Moreover, a plenary discussion of the case summaries was helpful for our ophthalmology residents. Incidentally, the case review resulted in the planning of additional examinations before the planned consultation, thereby improving the efficiency.

Importantly, our remote triaging arguably increased the safety of our population when compared with no care at all. Although this may sound as clear as day, the latter is an inconvenient reality for patients who spend weeks or months waiting for their appointments. In utopia, without restrictions on the amount of care we can deliver, we would happily invite everyone to our clinic for a specialist examination. In reality, scarcity of time and means demands alternative solutions to deal with the ever-increasing waiting lists for routine eye care that further soared during the pandemic. Access to eye care is of paramount importance, and TeleTriage is a novel approach to improve the access.

The biggest lesson learned during this project is that clinical decision-making is often possible *without* seeing patients in the clinic, especially during the routine follow-up of patients with chronic diseases. A judicious decision to cancel or postpone the consultations or refer patients to specialists could be made frequently based on the patients’ history, current complaints, home-assessed visual acuity, and knowledge of disease biology and epidemiology. However, at an almost equal rate, our ophthalmologists concluded that patient safety could be compromised when further delaying care and decided to maintain (or expedite) consultations at the clinic. The most commonly encountered reasons were the nature of the disease (eg, asymptomatic diseases such as progressive glaucoma), red flags in the case summary (eg, poorer visual acuity or vision symptoms), a lack of trust in the case summary (eg, inconsistencies or language barriers), or existing protocols mandating follow-up (eg, screening for hydroxychloroquine maculopathy or diabetic retinopathy). Naturally, important clinical findings such as ophthalmic examinations, intraocular pressure assessments, and optical coherence tomography imaging can only be assessed in person. For only a small fraction (4%) of the cases, the scheduled face-to-face consultation was changed into a telephonic consultation with their ophthalmologist, although it should be noted that treatment advice or feedback was often delivered via the TTT approach itself. These cases are de facto teleconsultations, paving the way for cancellation or postponement of consultations without compromising quality of care. In this way, while originally conceptualized for triaging, the TTT approach evolved into a telemedicine service that included the full assessments of patients *and* remote health care delivery.

The supposedly decreased *human interaction* between patients and their health care professionals is another ethical concern that is frequently introduced as an argument against telemedicine implementation. In this project, we experienced little resistance and no formal complaints from patients who were contacted by phone. The extraordinary situation of the pandemic eased the acceptance of this project, though we also consider the personalized approaches and tailored communication between the students and the patients a vital reason for success. A few patients opposed the final clinical decisions. This was most frequently encountered when patients had a long-standing relationship with our hospital and were referred to another eye care provider. Invariably, not all patients suitable for referral were referred. The data reported in this study reflect the final management rather than the initially proposed management. Differences between these 2 were not recorded; therefore, the true size of this effect could not be quantified.

Technology adoption is another challenge in the delivery of remote care. Not all patients are willing or able to use telemedicine services. In this project, most clinical decisions were based on the information gathered via phone. More or less all of our patients had access to telephone services, so we did not encounter technical difficulties while collecting these data. In addition, a platform for remote eye examinations was available to the team to collect quantifiable information about the visual function of the patients. As this service requires access to technology and basic digital skills, adoption issues arose. The proportion of internet access in the Netherlands has been reported to be the highest in Europe: 98% of households had direct internet access in 2019 [16]. Nevertheless, digital literacy is age associated and related to the technological competencies that were required during the life course [17,18]. Internet use is less common among the older generations [19]. Most of our ophthalmic population was older. Despite the high internet accessibility and—relatively high—digital literacy rate in the Netherlands compared with other European countries, the uptake of this eHealth application and its role in clinical decision-making was rather low for 2 reasons. First, the students did not invite all patients to perform the eye examination. Obviously, a quantifiable visual acuity outcome is not always essential for clinical decision-making, and unfamiliarity of new team members with the web-based platform reduces *professional adaptation*. More importantly, the lack of patient portal access and initial resistance of some patients to perform the computer-based test were the evident barriers that precluded the students from guiding the patients through the examination. Second, approximately half of the invited patients did not complete the eye examinations. Anecdotal telephonic feedback from patients who did not complete the test was collected by the research team (JC). Patients reported that the clinic’s patient portal environment was difficult to navigate. Frequently, there were problems with manually entering the test outcomes into the questionnaire. To a lesser degree, a lack of time or motivation was reported. Interestingly, the instructions for the eye test itself were reported to be clear. This is in line with a recently published study on cataract patients (mean age 70, SD 7 years) [20]. In-depth interviews and quantitative questionnaires based on Technology Acceptance Models identified an overall positive attitude toward the web-based eye test. We propose that better integration of this test into the patient portal will make it easier for patients to access the tool and, more importantly, will waive the need to manually enter one’s outcomes. Positive experiences are expected to increase staff confidence in inviting patients to perform the examination. Engaging patients in self-measurements can promote self-awareness, self-management, and ownership of one’s health and well-being. This complies with the transition to patient-centered care models, as reported in a World Health Organization report on eHealth implementation [21].

### Eye Care Delivery After the Pandemic

Changing demographics, increased technical possibilities, and a higher prevalence of systemic disorders with ocular manifestations (eg, diabetes) are expected to drastically increase the future demand not only for ophthalmic care [22,23] but also for other domains of health care [2]. In the Netherlands, it is estimated that by 2060, one in 3 people should be working in the health care industry to tackle these demands. As this is not feasible, alternative strategies are required to maintain health care accessible for all, such as prioritizing and improving efficiency [2]. Therefore, we propose that the TTT approach is highly valuable beyond the pandemic setting and of great interest for future purposes.

An important aspect of this project was that the practice pattern was preliminarily introduced within a short period. Our approach could be extended by enriching the remote monitoring platform with options for obtaining images remotely. In the United Kingdom, more evolved triaging workflows have been very successful in reducing hospital visits while maintaining communication, patient safety, and clinical quality, even before the pandemic [24-27]. Especially for retinal disorders, diagnosis and treatment rely increasingly on optical coherence tomography imaging devices rather than fundoscopy [28], which allows an asynchronous approach to diagnostics and clinical decision-making. Therefore, several eye clinics have started to refer patients to remote community clinics for obtaining these images. As not all screened retinas require treatment or further examination, this significantly reduces the burden of clinic visits. Interestingly, an added beneficial effect was higher attendance of diabetic retinopathy screening based on a telemedicine-based methodology when compared with conventional screening [29]. The combined approach of remote diagnostics with centralized asynchronous *augmented intelligence* clinical decision-making certainly holds promise for the future; this TeleTriage project provides important lessons in this regard. We hope that this manuscript inspires (young) colleagues to rethink how eye care is delivered and that it provides insights into how to become architects of this change. Future studies could focus on further exploring patients’ perspectives and cognitions on teletriaging, analyze clinical outcomes and safety aspects, and evaluate the cost-effectiveness of this telemedicine approach.

### Conclusions

In conclusion, our novel approach to remotely review cases and prioritize urgency has been successful in maintaining continuity of care despite the COVID-19 pandemic. The project evolved into a telemedicine service of great interest for future purposes, as it aligns with the current trends toward remote care delivery and reduces the burden of hospital visits. The asynchronous triaging allows efficient task allocation without compromising the quality of care, as medical specialists are responsible for the final clinical decisions. The paradox debated in this paper is that judicious clinical decision-making based on remotely collected data actually is possible, only if we as caregivers are willing to change our routines and cognitions regarding face-to-face care delivery. Patient acceptance of this novel method of care delivery is essential for success and is promoted by individual communication and tailored clinical decision-making (ie, patient-centered care). In addition, the triaging method has been highly valuable for educating future health care professionals in understanding the course of disease, communicating with patients, and clinical decision-making. This project serves as a proving ground for upcoming innovations in remote eye care delivery and could play a comparable role for other clinical domains.

## Abbreviations

CE: Conformité Européenne
EHR: electronic health record
GP: general practitioner
HU-UAS: HU University of Applied Sciences Utrecht
TTT: TeleTriageTeam
UMCU: University Medical Center Utrecht

## Data Availability

The data sets generated and analyzed during this study are not publicly available, as the paper is based on real-world clinical data and interim analyses were performed in the context of quality control and quality assurance, but are available from the corresponding author upon reasonable request.

## References

[1] (2020). Wachttijden polikliniek. Volksgezondheidenzorg.info.

[2] (2021). Kiezen voor houdbare zorg. Wetenschappelijke Raad voor het Regeringsbeleid.

[3] Wisse R, Mueller-Schotte S, Hortensius V, Imhof S (2021). Oogkwalen op afstand beoordelen gaat prima. Medisch Contact (Bussum).

[4] Wisse RP, Muijzer MB, Cassano F, Godefrooij DA, Prevoo YF, Soeters N (2019). Validation of an independent web-based tool for measuring visual acuity and refractive error (the manifest versus online refractive evaluation trial): prospective open-label noninferiority clinical trial. J Med Internet Res.

[5] Muijzer MB, Claessens JL, Cassano F, Godefrooij DA, Prevoo YF, Wisse RP (2021). The evaluation of a web-based tool for measuring the uncorrected visual acuity and refractive error in keratoconus eyes: a method comparison study. PLoS One.

[6] Claessens J, van Egmond J, Wanten J, Bauer N, Nuijts R, Wisse R (2023). The accuracy of a web-based visual acuity self-assessment tool performed independently by eye care patients at home: method comparison study. JMIR Form Res.

[7] Wanten JC, Bauer NJ, Claessens JL, van Amelsfort T, Berendschot TT, Wisse RP, Nuijts RM (2023). Evaluation of a visual acuity eHealth tool in patients with cataract. J Cataract Refract Surg.

[8] (2021). Gezondheidszorg. Rijksoverheid: Gezondheid en zorg.

[9] (2005). CCMO-notitie definitie medisch-wetenschappelijk onderzoek. The Central Committee on Research Involving Human Subjects.

[10] Mathieu E, Ritchie H, Rodés-Guirao L, Appel C, Giattino C, Hasell J, Macdonald B, Dattani S, Beltekian D, Ortiz-Ospina E, Roser M (2020). Coronavirus pandemic (COVID-19). Our World in Data.

[11] (2021). Analyse van de gevolgen van de coronacrisis voor verwijzingen medisch-specialistische zorg en inzichten uit Zorgbeeld - 19 januari 2021. Nederlandse Zorg Autoriteit.

[12] (2022). Juiste zorg op de juiste plek. Federatie Medisch Specialisten.

[13] van den Heuvel JF, Lely AT, Huisman JJ, Trappenburg JC, Franx A, Bekker MN (2020). SAFE@HOME: digital health platform facilitating a new care path for women at increased risk of preeclampsia - a case-control study. Pregnancy Hypertens.

[14] Smit K (2021). Nieuw onderzoek naar thuis zuurstofsaturatie meten bij COVID-19-patiënten. Huisarts Wet.

[15] Zorg D (2022). Langer thuis met CovidTherapy@home en Early@home. UMC Utrecht.

[16] (2022). Digital economy and society statistics - households and individuals. Eurostat.

[17] Oh SS, Kim K, Kim M, Oh J, Chu SH, Choi J (2021). Measurement of digital literacy among older adults: systematic review. J Med Internet Res.

[18] Chesser A, Burke A, Reyes J, Rohrberg T (2016). Navigating the digital divide: a systematic review of eHealth literacy in underserved populations in the United States. Inform Health Soc Care.

[19] (2021). How popular is internet use among older people?. Eurostat.

[20] Claessens JL, Maats EP, Iacob ME, Wisse RP, Jongsma KR (2023). Introducing e-health technology to routine cataract care: patient perspectives on a web-based eye test for postoperative telemonitoring. J Cataract Refract Surg.

[21] (2016). From innovation to implementation: eHealth in the WHO European region. World Health Organization.

[22] Williams LB, Prakalapakorn SG, Ansari Z, Goldhardt R (2020). Impact and trends in global ophthalmology. Curr Ophthalmol Rep.

[23] Kotecha A, Turner S, Vasilakis C, Utley M, Fulop N, Azuara-Blanco A, Foster PJ (2014). Improving care and increasing efficiency-challenges in the care of chronic eye diseases. Eye (Lond).

[24] Kern C, Kortuem K, Hamilton R, Fasolo S, Cai Y, Balaskas K, Keane P, Sim D (2019). Clinical outcomes of a hospital-based teleophthalmology service: what happens to patients in a virtual clinic?. Ophthalmol Retina.

[25] Sim D (2019). Glimpses of the future: virtual clinics at Moorfields. The Ophthalmologist.

[26] Kortuem K, Fasler K, Charnley A, Khambati H, Fasolo S, Katz M, Balaskas K, Rajendram R, Hamilton R, Keane PA, Sim DA (2018). Implementation of medical retina virtual clinics in a tertiary eye care referral centre. Br J Ophthalmol.

[27] Faes L, Fu DJ, Huemer J, Kern C, Wagner SK, Fasolo S, Hamilton R, Egan C, Balaskas K, Keane PA, Bachmann LM, Sim DA (2021). A virtual-clinic pathway for patients referred from a national diabetes eye screening programme reduces service demands whilst maintaining quality of care. Eye (Lond).

[28] Eladawi N, Elmogy MM, Ghazal M, Helmy O, Aboelfetouh A, Riad A, Schaal S, El-Baz A (2018). Classification of retinal diseases based on OCT Images. Front Biosci (Landmark Ed).

[29] Mansberger SL, Sheppler C, Barker G, Gardiner SK, Demirel S, Wooten K, Becker TM (2015). Long-term comparative effectiveness of telemedicine in providing diabetic retinopathy screening examinations: a randomized clinical trial. JAMA Ophthalmol.

